# Spatial network and driving factors of low-carbon patent applications in China from a public health perspective

**DOI:** 10.3389/fpubh.2023.1121860

**Published:** 2023-02-17

**Authors:** Feng Hu, Liping Qiu, Yang Xiang, Shaobin Wei, Han Sun, Hao Hu, Xiayan Weng, Lidan Mao, Ming Zeng

**Affiliations:** ^1^Institute of International Business and Economics Innovation and Governance, Shanghai University of International Business and Economics, Shanghai, China; ^2^Institute of International Business, Zhejiang Gongshang University, Hangzhou, China; ^3^The Second Bethune Clinical Medical College, Jilin University, Changchun, China; ^4^China Center for Economic Research, East China Normal University, Shanghai, China; ^5^Institute of Digital Economy and Green Development, Chifeng University, Chifeng, China; ^6^School of Economics, Shanghai University, Shanghai, China; ^7^School of MBA, Zhejiang Gongshang University, Hangzhou, China; ^8^Hangzhou Business School, Zhejiang Gongshang University, Hangzhou, China; ^9^School of Tourism Management, Zhangjiajie Institute of Aeronautical Engineering, Zhangjiajie, China

**Keywords:** public health, low-carbon patent application, social network, spatial correlation structure, urban agglomeration

## Abstract

**Introduction:**

The natural disasters and climate anomalies caused by increasing global carbon emissions have seriously threatened public health. To solve increasingly serious environmental pollution problems, the Chinese government has committed itself to achieving the goals of peak carbon emissions and carbon neutrality. The low-carbon patent application is an important means to achieve these goals and promote public health.

**Methods:**

This study analyzes the basic situation, spatial network, and influencing factors of low-carbon patent applications in China since 2001 at the provincial and urban agglomeration levels using social network analysis based on data from the Incopat global patent database.

**Results:**

The following findings are established. (1) From the number of low-carbon patent applications, the total number of low-carbon patent applications in China increased year by year, while the number of applications in the eastern region was larger than those in the central and western regions, but such regional differences had been decreasing. (2) At the interprovincial level, low-carbon patent applications showed a complex and multithreaded network structure. In particular, the eastern coastal provinces occupied the core position in the network. The weighted degree distribution of China's interprovincial low-carbon patent cooperation network is affected by various factors, including economic development, financial support, local scientific research level, and low-carbon awareness. (3) At the urban agglomeration level, the eastern coastal urban agglomerations showed a radial structure with the central city as the core. Urban innovation capability, economic development, low-carbon development awareness, level of technology import from overseas, and informatization level are highly correlated with the weighted degree of low-carbon cooperation networks of urban agglomerations.

**Discussion:**

This study provides ideas for the construction and governance of low-carbon technology innovation system and perspectives for theoretical research on public health and high-quality development in China.

## 1. Introduction

Climate warming has become a global concern. Countries around the world have taken measures to address this issue, including successively signing three international laws, namely, the United Nations Framework Convention on Climate Change, the Kyoto Protocol, and the Paris Agreement, to establish a global climate governance system (1). The Chinese government attaches great importance to this issue and has committed itself to achieving the goals of peak carbon emissions and carbon neutrality by 2030 and 2060, respectively (2, 3). Low-carbon technology is an important way to achieve these goals. Low-carbon patents are an innovative form of low-carbon technology and enable accurate measurement of the research situation of low-carbon technology in various regions (4). Since the development of low-carbon innovation in various regions of China is strongly influenced by regional economic and other factors, it leads to spatial heterogeneity in low-carbon technology innovation, which poses a challenge to the formation of cross-regional cooperation networks. Therefore, it is necessary to analyze the overall network structure and evolution of low-carbon collaborative innovation and identify the key roles of different regions in the network, as well as the influencing factors affecting regional low-carbon technologies, in order to improve the regional collaboration of low-carbon innovation, find influential partners for local governments' low-carbon innovation activities, and provide objective references for public health policy formulation (5).

In recent years, patent cooperation networks have become a popular Research Topic, in which the analysis of data related to patent information, joint patent applications, and collaborative papers are the main data sources for technological innovation activities (6–8). Numerous studies have been undertaken on patent cooperation networks in terms of different disciplines and perspectives using social network analysis and other methods based on data from world-renowned patent databases, such as the databases of the USPTO and the EPO and the Incopat database. For example, the formation, evolution, structure, and characteristics of patent cooperation networks in industries such as solar cells (9, 10), nanotechnology (11, 12), and measurement and chemistry (13) were investigated by analyzing centrality, structural holes, network density, and other indicators (14–19).

In recent years, the formation mechanism of the spatial network of patent applications has begun to receive considerable attention. It has been reported that geographic distance, technological relevance, small-world effects, social proximity, and cooperative relationships all have an impact on network formation (20–24). A literature review reveals that the spatial network of patent applications has rarely been investigated from the perspective of regions and urban agglomerations, and it is even rarer to focus on low-carbon patents. Moreover, previous studies only focus on a single level, such as country, province, urban agglomeration, urban area, or rural area (25–28). Few studies have comprehensively examined the spatial network of low-carbon patent applications and the influencing factors from the perspective of regions, provinces, and urban agglomerations.

At present, China is in a critical period of low-carbon transformation (29). In addition, it may be difficult to control the global impact of the COVID-19 pandemic in a short period of time. Therefore, it is worth considering how to achieve the goals of peak carbon emissions and carbon neutrality in an orderly manner in the post-pandemic period. To this end, this study aims to investigate the spatial characteristics of low-carbon technologies in China at the levels of urban agglomerations, regions, and provinces using low-carbon patent cooperation data to identify problems from a new perspective and help clarify the current technology accumulation in this field in China. On this basis, strategies to optimize the low-carbon patent cooperation network in the post-pandemic period are proposed to provide patent information support for scientific work in the low-carbon field and to provide a theoretical basis for patented technology development strategies.

This study provides the following marginal contributions. First, this study expands the theoretical system of the geography of innovation in a vertical three-dimensional way through social network analysis. Second, this study analyzes the innovation functions and roles of cities in the networks of urban agglomerations, regions, and provinces, strengthening the theoretical research of urban geography. Third, the conclusions may provide a strategic basis for the government and related functional departments to innovate and develop low-carbon technologies.

The remainder of the paper is structured as follows: Section 2 describes the data and methodology. Section 3 analyzes the spatiotemporal evolution of low-carbon patent applications in China at the national level. Sections 4 and 5 analyze the spatial structure of the patent network and the driving factors at the provincial level. Sections 6 and 7 present a discussion at the level of urban agglomerations. Section 8 presents the conclusions, implications, potential contributions, and limitations.

## 2. Data and methodology

### 2.1. Subjects

This study covers 31 provinces/municipalities in three regions of China (except Hong Kong, Macau, and Taiwan) and five major urban agglomerations along the eastern coast (Table 1) (30).

**Table 1:** Description of the correspondence of the study subjects.

**Region**	**Province**	**Urban agglomeration**	**City**
East	Beijing, Tianjin, Hebei, Liaoning, Shanghai, Jiangsu, Zhejiang, Fujian, Shandong, Guangdong, Hainan	Beijing-Tianjin-Hebei (BTH) region	Baoding, Beijing, Cangzhou, Chengde, Langfang, Qinhuangdao, Shijiazhuang, Tangshan, Tianjin, Zhangjiakou
Central	Shanxi, Jilin, Heilongjiang, Anhui, Jiangxi, Henan, Hubei, Hunan	Pearl River Delta (PRD)	Guangzhou, Shenzhen, Foshan, Dongguan, Zhaoqing, Jiangmen, Zhuhai, Huizhou, Zhongshan
West	Sichuan, Chongqing, Guizhou, Yunnan, Tibet, Shaanxi, Gansu, Qinghai, Ningxia, Xinjiang, Guangxi, Inner Mongolia	Yangtze River Delta (YRD)	Changzhou, Hangzhou, Huzhou, Jiaxing, Nanjing, Nantong, Ningbo, Shanghai, Shaoxing, Suzhou, Taizhou, Taizhou, Wuxi, Yangzhou, Zhenjiang, Zhoushan
		Shandong Peninsula (SP)	Binzhou, Dezhou, Dongying, Jinan, Liaocheng, Qingdao, Rizhao, Tai'an, Weihai, Weifang, Yantai, Zibo
		Western Taiwan Straits (WTS) Economic Zone	Chaozhou, Fuzhou, Ningde, Putian, Quanzhou, Xiamen, Shantou, Zhangzhou, Wenzhou, Jieyang, Shanwei

### 2.2. Methods and data

#### 2.2.1. Data

In 2013, the United States Patent and Trademark Office (USPTO) and the European Patent Office (EPO) jointly launched the CPC-Y02 patent classification system for technologies or applications for mitigation or adaptation against climate change. Due to the structural and systemic advantages of this system, it has been extensively used in the research of low-carbon patent-related issues in recent years (31, 32).

Low-carbon patent application data are from the Incopat global patent database. Specifically, patents in the CPC-Y02 class were retrieved from the Incopat database on August 26, 2022.

#### 2.2.2. Methods

Social network analysis is a quantitative analysis method developed from mathematical methods and graph theory and in recent years has become one of the most widely used methods in sociology and economics (33). This study uses social network analysis and related theories to analyze the structural characteristics, network pattern evolution, and driving factors of China's low-carbon patent application network. The indicators involved in this study are as follows.

Centrality: This measures the node control in the entire factor network and is defined as follows:


(1) $$\begin{array}{l} C_{D}\left(n_{i}\right)=d\left(n_{i}\right)=\sum_{j}X_{ij}=\sum_{i}X_{ji},{C^{\prime }}_{D}\left(n_{i}\right)=\frac{d\left(n_{i}\right)}{g-1} \end{array}$$

Where *C*_*D*_(*n*_*i*_) is the absolute centrality of node *i*; *X*_*ij*_ is 0 or 1, indicating whether nodes *j* and *i* are related or not; and *g* is the number of network nodes.

Closeness centrality measures how close a node is to all other nodes, reflecting its control over other nodes.


(2) $$\begin{array}{l} CC_{i}=\frac{g-1}{\sum_{j=1,j\neq i}^{N}d_{ij}} \end{array}$$

Where *d*_*ij*_ is the number of steps in the shortest path between nodes *i* and *j*.

Betweenness centrality measures the degree to which a node is located at the center of other nodes, thus reflecting a state's ability to control the channels and mediate the flow of energy in the network. Assuming that the number of shortest paths between nodes *j* and *k* is *g*_*jk*_ and the number of shortest paths between nodes *j* and *k* passing through node *i* is *g*_*jk*_(*i*), the ability of node *i* to control the association between nodes *j* and *k* can be defined as $b_{jk}=\frac{g_{jk}\left(i\right)}{g_{jk}}$.


(3) $$\begin{array}{l} BC_{i}=\frac{2\sum_{j}^{n}\sum_{k}^{n}b_{jk}\left(i\right)}{N^{2}-3N+2},\text{ where }j\neq k\;\neq i\text{ and }j<k \end{array}$$

Weighted degree: This measures the weight of low-carbon patent cooperation that occurs at a node. A higher weighted degree value means a higher weight of low-carbon patent cooperation at the node. The weighted degree can be divided into weighted in-degree and out-degree. It is calculated as follows:


(4) $$\begin{array}{l} C_{w}\left(i\right)=\sum_{j=1}^{N}W_{ij}+\sum_{j=1}^{N}W_{ji} \end{array}$$

Where *C*_*w*_(*i*), $\overset{N}{\underset{j=1}{\sum }}W_{ij}$, and $\overset{N}{\underset{j=1}{\sum }}W_{ji}$ are the weighted degree, weighted out-degree, and weighted in-degree of node *i*, respectively.

GeoDetector: GeoDetector is a spatial analysis model used to assess the relationship between a geographical attribute and its explanatory factors. It is widely used to investigate the influencing factors of natural and socioeconomic phenomena. GeoDetector requires only a few preconditions and has obvious advantages when dealing with mixed-type data. The factor detector in GeoDetector was used to assess the explanatory power of each influencing factor and its changes in the centrality of China's interprovincial low-carbon patent cooperation network (34). The factor detector is expressed as follows:


(5) $$\begin{array}{l} q=1-\frac{\sum_{h=1}^{L}\sigma_{h}^{2}N_{h}}{N\sigma^{2}} \end{array}$$

Where *q* is the detection capability of an influencing factor for the centrality of China's interprovincial low-carbon patent cooperation network; *h* = 1…; L is the classification of each factor of the variable; σ^2^ is the total variance of the centrality of the interprovincial low-carbon patent cooperation network; $\sigma_{h}^{2}$ is the variance of the interprovincial low-carbon patent cooperation network; N is the number of provinces in China; and Nh is the number of types of influencing Factor *X*. The value range of q is (0, 1). The larger *q* is, the greater the influence of this factor on interprovincial low-carbon patent cooperation.

Correlation coefficient: This measures how closely two phenomena (elements) are correlated. Due to factors such as network capital, there are differences in the number of low-carbon patent applications in different provinces and cities. The relationship of the number of low-carbon patent applications with the effective size, constraint, closeness, betweenness, and weighted degree of the province was examined by linear correlation analysis. The correlation coefficient is calculated as follows:


(6) $$\begin{array}{l} \gamma =\frac{\sum \left(x-\bar{x}\right)\left(y-\bar{y}\right)}{\sqrt{{\left(x-\bar{x}\right)}^{2}{\left(y-\bar{y}\right)}^{2}}} \end{array}$$

Where γ is the correlation coefficient; x and y are two sets of variables; and $\bar{x}\;and\;ȳ$ are the means of the variables. The value of γ is between −1 and 1. γ > 0 indicates a positive correlation, and γ < 0 indicates a negative correlation. The larger the absolute value of γ is, the greater the correlation.

Low-carbon search index: The low-carbon search indices of prefecture-level cities or provinces from January 1 to December 31, 2021, were collected from the Baidu Index website using the keyword “low-carbon” to reflect online attention to the low-carbon field.

Block model: The block model is a method of analyzing the node location in a network (35, 36). Wasserman and Faust (37) created a method for evaluating the relationship between positions within a network. According to the classifications of previous studies, four blocks are defined according to position: bidirectional spillover, main benefit, broker, and main spillover blocks (38).

GRA: This analysis is based on gray system theory. The parent sequence is the centrality of the urban low-carbon patent cooperation network (39). The subsequences are the number of patent applications, per capita GDP, low-carbon search index, total volume of post and telecommunications business, and actually utilized value of foreign direct investment, representing 5 influencing factors. The correlation coefficients were calculated by non-dimensionalizing the original data. DPS software was used for data processing and analysis to reduce the difficulties caused by complex modeling and the large amount of data (40, 41).

## 3. Spatiotemporal evolution of low-carbon patent applications

### 3.1. Number of low-carbon patent applications

**Figure 1:**
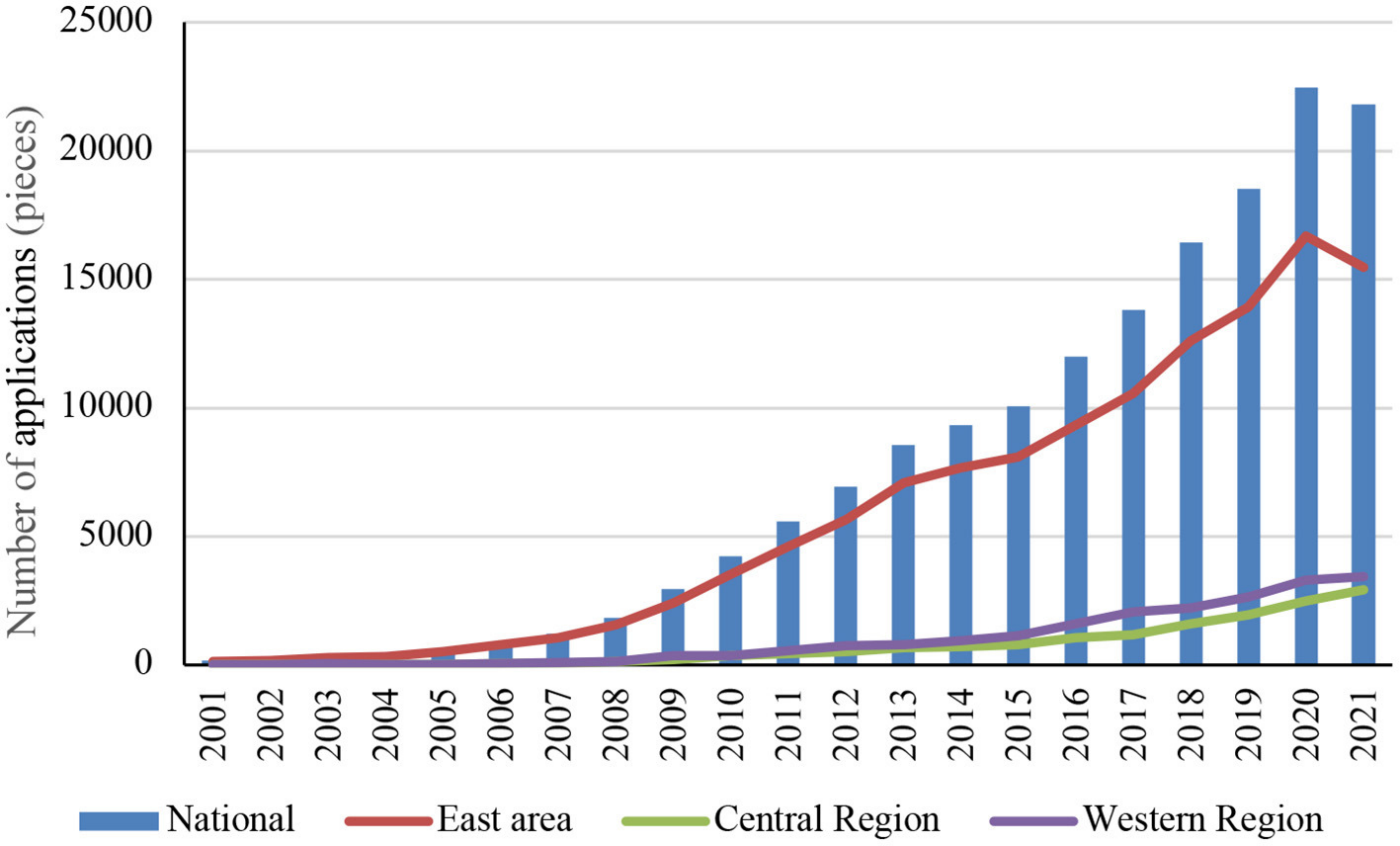
Number of low-carbon patent applications nationwide and in the eastern, central, and western regions.

As shown in Figure 1, the number of China's low-carbon patent applications increased year by year until 2021, when it decreased due to the COVID-19 pandemic. At the regional level, the number of applications in the eastern region was much higher than that in the central and western regions combined. The number of patent applications in the eastern region as a percentage of the national total increased from 85.09% in 2001 to 89.03% in 2006 and then decreased to 70.83% in 2021. The number of patent applications in the central and western regions as a percentage of the national total decreased from 6.83 and 8.07% in 2001 to 5.15 and 5.82% in 2006 and then increased to 13.41 and 15.76% in 2021, respectively.

### 3.2. Spatial differences in low-carbon patent applications

As shown in Table 2, there are obvious regional differences in the number of low-carbon patent applications in China. Overall, the differences in the number of low-carbon patent applications both within and between the three regions continued to decrease. The interprovincial Gini coefficient decreased from 0.9333 in 2001 to 0.5791 in 2021, indicating a decreasing difference in the number of low-carbon patent applications between provinces. At the regional level, the Gini coefficient within the eastern region continued to decrease, indicating a decreasing internal difference. The Gini coefficients within the central and western regions increased and then decreased, peaking in 2006 and 2011, respectively.

**Table 2:** The Gini coefficients of low-carbon patent applications nationwide and in the eastern, central, and western regions.

**Year**	**Gini coefficient**	**Intraregional difference**		
		**East**	**West**	**Central**
2001	0.9333	0.8390	0.4722	0.1481
2006	0.8853	0.6944	0.3864	0.6296
2011	0.8219	0.5645	0.6452	0.1774
2016	0.7999	0.5390	0.2313	0.1240
2021	0.5791	0.1051	0.2623	0.0118

### 3.3. Spatial trends of low-carbon patent applications in provinces

**Figure 2:**
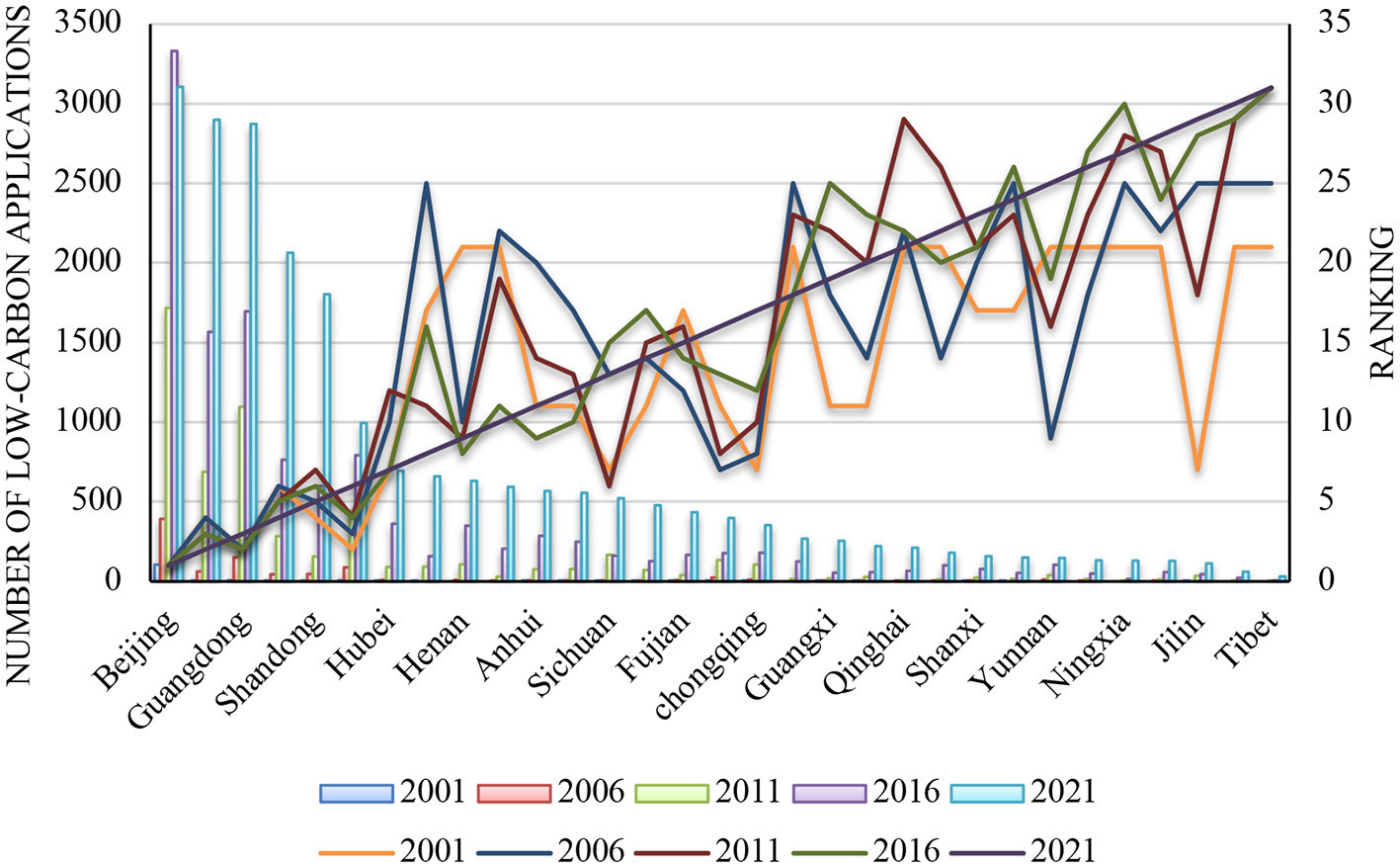
Number and ranking of low-carbon patent applications of the 31 provinces/municipalities.

As shown in Figure 2, two characteristics are present in the number of low-carbon patent applications in China's 31 provinces/municipalities due to local economic development, scientific research, and policies. First, there were great changes in the number of low-carbon patent applications. Most provinces/municipalities showed changes in both the number of low-carbon patent applications and the ranking. In particular, Beijing was always ranked first in the number of low-carbon patent applications. Jiangsu rose from fourth in 2001 to second in 2021. Guangdong was always ranked in the top three. Fujian, Beijing, Guangxi, and Hunan showed small changes in the ranking by the number of low-carbon applications. Shandong, Tianjin, Jiangsu, Zhejiang, Hubei, Anhui, Qinghai, Henan, Jiangxi, Shaanxi, and Hebei rose in rankings, while the rest of the provinces dropped. Second, the number of low-carbon patent applications varied significantly between provinces. For example, the number of low-carbon patent applications in Beijing, Jiangsu, Guangdong, Zhejiang, and Shandong exceeded 1,000 in 2021, whereas it was <500 in Heilongjiang, Jilin, and Liaoning in Northeast China.

## 4. Evolution of the low-carbon patent application spatial network at the province level

### 4.1. General network characteristics

The overall network characteristics were measured using Gephi and Ucinet. First, the overall network density was increased, as shown in Table 3. The network density increased from 0.133 in 2001 to 0.847 in 2021, indicating that China has formed an effective low-carbon cooperation network as a whole. There are obvious spillover correlations of patent cooperation among all provinces, and the degree of such cooperation is increasing. Second, the evolution trend of the average shortest path length and the clustering coefficient showed increasing clustering of China's low-carbon patent cooperation network. The average path length of the network shows an upward and then downward trend over time, with a mean value of 1.671. Third, as revealed by the time series, the average degree increased from 2.667 in 2001 to 25.419 in 2021, showing an overall increase. This indicates that the provinces have an increasing influence on China's low-carbon patent cooperation network.

**Table 3:** Overall characteristics of China's low-carbon patent cooperation network.

**Year**	**Number of nodes**	**Number of edges**	**Network density**	**Average clustering coefficient**	**Average path length**	**Average degree**
2001	21	28	0.133	0.702	1.965	2.667
2006	26	53	0.163	0.479	2.138	4.077
2011	31	169	0.363	0.663	1.699	10.903
2016	31	278	0.598	0.746	1.402	17.935
2021	31	394	0.847	0.890	1.153	25.419

### 4.2. Individual network characteristics

The weighted degree reflects the status of each province in China's low-carbon patent cooperation network to some extent. As shown in Table 4, the weighted degrees of Beijing, Shanghai, Guangdong, Jiangsu, Zhejiang, and Tianjin along the eastern coast were ranked among the top 10 in 2001. In 2011, the network pattern changed. Specifically, Shanxi, Hubei, Shandong, Anhui, and Sichuan dropped out of the top ten, whereas Fujian, Henan, and Hebei had increased weighted degrees and broke into the top ten nationwide. In 2021, Shanghai dropped from second to fifth due to the COVID-19 pandemic. Meanwhile, Jiangsu broke into the top three, Liaoning, Fujian and Henan dropped out of the top 10, and Shandong returned to the top ten.

**Table 4:** Top 10 provinces/municipalities in China's low-carbon cooperation network in terms of weighted degree in 2001–2021.

**2001**	**Weighted degree**	**2011**	**Weighted degree**	**2021**	**Weighted degree**
Beijing	77	Beijing	2,025	Beijing	10,051
Shanghai	56	Shanghai	925	Jiangsu	5,068
Guangdong	21	Guangdong	894	Guangdong	4,361
Shanxi	16	Jiangsu	681	Zhejiang	2,464
Hubei	7	Liaoning	408	Shanghai	2,243
Shandong	7	Tianjin	355	Shandong	1,861
Jiangsu	5	Zhejiang	290	Hebei	1,773
Liaoning	4	Fujian	286	Tianjin	1,614
Zhejiang	4	Henan	208	Hunan	1,598
Anhui	3	Hebei	178	Shaanxi	1,360

Betweenness centrality reflects the shortest cooperation path between provinces in China's low-carbon patent cooperation network. The higher it is, the greater the control of the province. As shown in Table 5, Beijing, Shanghai, Guangdong, Jiangsu, and Hubei ranked high in terms of betweenness centrality from 2001 to 2020, indicating that these provinces/municipalities are at the core of China's low-carbon patent cooperation network.

**Table 5:** Top 10 provinces/municipalities in China's low-carbon cooperation network in terms of betweenness and closeness centrality in 2001–2021.

**2001**	**Betweenness**	**2011**	**Betweenness**	**2021**	**Betweenness**	**2001**	**Closeness**	**2011**	**Closeness**	**2021**	**Closeness**
Beijing	127.333	Beijing	59.143	Beijing	4.214	Hunan	1.000	Beijing	0.882	Beijing	1.000
Guangdong	28.833	Shanghai	39.889	Guangdong	4.214	Chongqing	1.000	Shanghai	0.811	Jiangsu	1.000
Hubei	5.667	Jiangsu	38.026	Hubei	4.214	Beijing	0.900	Jiangsu	0.811	Guangdong	1.000
Shanghai	1.833	Shaanxi	30.520	Jiangsu	4.214	Guangdong	0.621	Guangdong	0.750	Shanghai	1.000
Shanxi	1.833	Liaoning	25.014	Shaanxi	4.214	Shanghai	0.563	Liaoning	0.667	Shaanxi	1.000
Jiangsu	0.500	Sichuan	18.824	Shanghai	4.214	Shanxi	0.563	Zhejiang	0.667	Hubei	1.000
Anhui	0.000	Guangdong	18.456	Chongqing	3.944	Hubei	0.545	Henan	0.667	Zhejiang	0.968
Gansu	0.000	Henan	13.831	Sichuan	3.607	Sichuan	0.529	Hunan	0.667	Shandong	0.968
Hebei	0.000	Zhejiang	13.767	Shandong	3.602	Jiangsu	0.514	Shaanxi	0.667	Sichuan	0.968
Jiangxi	0.000	Hubei	12.936	Tianjin	3.559	Shandong	0.500	Sichuan	0.652	Anhui	0.968

Closeness centrality reflects the sum of low-carbon patent cooperation distances between provinces. The higher it is, the closer the province is to other provinces in the low-carbon patent cooperation network. Closeness centrality also indicates the degree of independence of nodes. In 2001, Hunan, Chongqing, Beijing, Guangdong, Shanghai, Shanxi, Hubei, Sichuan, Jiangsu, and Shandong ranked high in terms of closeness centrality, as shown in Table 5. Except for Shaanxi, Zhejiang, and Anhui, the top 10 provinces in 2021 are the same as those in 2001.

### 4.3. Segmentation of the low-carbon patent cooperation network

The low-carbon patent cooperation network is segmented using the CONCOR block model method to investigate the spatial clustering of provincial patent cooperation in China. Specifically, the maximum segmentation density is set to 2, and the convergence criterion is 0.2. The Chinese low-carbon patent cooperation network in 2021 is divided into bidirectional spillover, main benefit, and broker blocks through two iterations. The segmentation results are shown in Table 6. The provinces included in each block are shown in Table 7.

**Table 6:** Segmentation of China's low-carbon patent cooperation network in 2021.

**2021**	**Number of members**	**Number of relations received**			**Number of relations sent**			**Proportion of expected internal relations (%)**	**Proportion of actual internal relations (%)**	**Block**
		**Inside the block**	**Outside the block**	**Total**	**Inside the block**	**Outside the block**	**Total**			
Block 1	9	60	148	208	60	146	206	26.67	29.13	Main benefit block
Block 2	17	183	170	353	183	173	356	53.33	51.40	Bidirectional spillover block
Block 3	3	6	81	87	6	80	86	6.67	3.49	Broker block
Block 4	2	2	30	32	2	30	32	3.33	6.25	Main benefit block

**Table 7:** Provinces/municipalities in each block of China's low-carbon patent cooperation network in 2021.

**Block**	**Provinces/municipalities**
Block 1	Anhui, Jiangxi, Fujian, Tianjin, Hubei, Hunan, Tibet, Jiangsu, and Zhejiang
Block 2	Guizhou, Gansu, Qinghai, Henan, Jilin, Shaanxi, Liaoning, Chongqing, Inner Mongolia, Ningxia, Shandong, Xinjiang, Shanxi, Hebei, Sichuan, Heilongjiang, and Yunnan
Block 3	Beijing, Guangdong, and Shanghai
Block 4	Hainan and Guangxi

In terms of the composition of block members, Block 1 received and sent 208 and 206 relations in 2021, respectively. The proportion of expected internal relations (26.67%) is smaller than that of the actual internal relations (29.13%). A total of 32 relations were received and sent by Block 4. The proportion of expected internal relations (3.33%) is smaller than that of actual internal relations (6.25%). Thus, these two blocks are both main benefit blocks. They are largely driven by patent cooperation in other provinces. Block 3, including Beijing, Guangdong, and Shanghai, received and sent a total of 87 and 86 relations, respectively. The proportion of expected internal relations (6.67%) is larger than that of actual internal relations (3.49%). Hence, it is a broker block. This block has played an important role in driving the low-carbon patent cooperation of other blocks. Block 2, represented by Chongqing, Shandong, and Sichuan, received and sent a total of 353 and 356 relations, respectively. The proportion of expected internal relations (53.33%) is larger than that of actual internal relations (51.40%). Hence, it is a bidirectional spillover block. Provinces within this block have strong low-carbon patent cooperation, which not only has a spatial spillover effect on other provinces inside the block but also drives the low-carbon patent cooperation of provinces outside the block.

**Figure 3:**
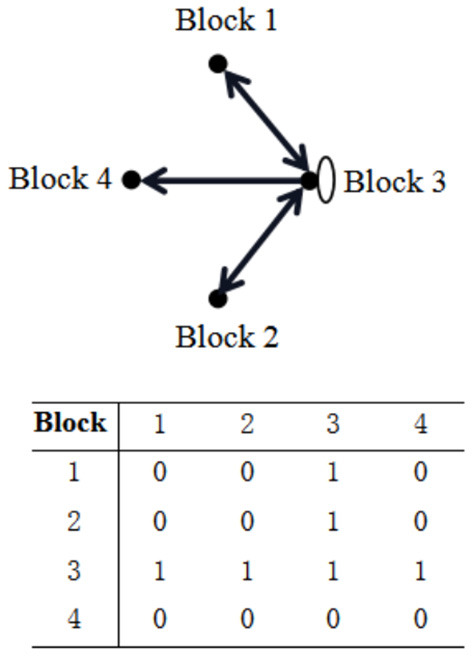
Relationship between the blocks of China's low-carbon cooperation network in 2021 and the image matrix.

In addition, an image matrix is generated using the density criterion to describe the relationship between the blocks. The values in the density matrix for each year that are greater than the overall network density are replaced with 1, and those smaller than the overall network density are replaced with 0. In this way, the relationship between the blocks at each stage and the image matrix are obtained, as shown in Figure 3. In 2021, the strongest internal relations were observed in Block 3. In addition to close patent cooperation between its members, Block 3 sent and received a large number of relations to and from Blocks 1 and 2. Except for receiving relations from Block 3, Block 4 rarely sent and received relations to and from other blocks. Relations within Block 4 were also scarce. There is a clubbing effect between the blocks. Therefore, block members with sparse relations should focus on strengthening patent cooperation with external core block members to avoid being “outliers” to ensure their position in the low-carbon patent cooperation network.

### 4.4. Core-periphery structure in a low-carbon patent cooperation network

The coreness of each province was calculated using Ucinet. Provinces with coreness values >0.2 were included in the core area. The distribution of provinces in the core area is shown in Table 8. From 2001 to 2021, Beijing held the top spot in China's low-carbon patent cooperation network in terms of coreness. Jiangsu has jumped from the semiperipheral area to the core area since 2016. Henan and Zhejiang were among the core provinces in 2016 but were replaced by Guangdong and Hebei in 2021.

**Table 8:** Core provinces in China's low-carbon patent cooperation network each year.

**Year**	**Core province**
2001	Beijing
2006	Beijing
2011	Beijing
2016	Beijing, Jiangsu, Henan, and Zhejiang
2021	Beijing, Guangdong, Hebei, and Jiangsu

### 4.5. Interregional relations in a low-carbon patent cooperation network

China was divided into eastern, central, and western regions to calculate the basic statistical characteristics of the network using Gephi. As seen from Table 9, the interregional low-carbon patent cooperation network improved significantly over time. The number of east-central, east-west, and central-west nodes increased from 10, 8, and 2 in 2001 to 19, 22, and 21 in 2021, respectively, and the number of edges increased from 10, 7, and 1 in 2001 to 84, 103, and 78 in 2021, respectively. In terms of network connectivity, the average degree increased from 2, 1.75, and 1 in 2001 to 8.842, 9.364, and 7.429 in 2021, respectively, and the average weighted degree increased from 6.2, 3, and 2 in 2001 to 474, 363.091, and 75.143 in 2021, respectively. At the intraregional level, the number of east-east, central-central, and west-west nodes increased from 8, 0, and 0 in 2001 to 10, 9, and 12 in 2021, respectively, and the number of edges increased from 10, 0, and 0 in 2001 to 45, 32, and 52 in 2021, respectively. In terms of network connectivity, the average degree increased from 2.5, 0, and 0 in 2001 to 9, 7.111, and 8.667 in 2021, respectively, and the average weighted degree increased from 16.5, 0, and 0 in 2001 to 2015.6, 83.778, and 115.333 in 2021, respectively. Overall, the interregional low-carbon patent cooperation network is not only more closely connected but also has stronger connection strength than the intraregional interprovincial cooperation network, except for east-east cooperation.

**Table 9:** Statistical characteristics of the interprovincial low-carbon patent cooperation network between the eastern, central, and western regions of China.

**Region**	**Year**	**Number of nodes**	**Number of edges**	**Average degree**	**Average weighted degree**
East-east	2001	8	10	2.5	16.5
	2011	10	31	6.2	437.6
	2021	10	45	9	2015.6
Central-central	2001	**-**	**-**	**-**	**-**
	2011	9	11	2.444	8.889
	2021	9	32	7.111	83.778
West-west	2001	**-**	**-**	**-**	**-**
	2011	11	11	2	12.727
	2021	12	52	8.667	115.333
East-west	2001	8	7	1.75	3
	2011	19	43	4.526	44.526
	2021	22	103	9.364	363.091
East-central	2001	10	10	2	6.2
	2011	19	53	5.579	102.842
	2021	19	84	8.842	474
Central-west	2001	2	1	1	2
	2011	16	20	2.5	8.5
	2021	21	78	7.429	75.143

## 5. Influencing factors of interprovincial low-carbon patent cooperation network

### 5.1. Selection of influencing factors

The correlation coefficients between the weighted degree of China's interprovincial low-carbon patent cooperation network and the influencing factors were calculated with the weighted degree of the network in 2021 as the dependent variable and GDP per capita, per capita disposable income of residents, and the value added of the secondary and tertiary industries as a percentage of GDP as independent variables. The data are from the China Statistical Yearbook.

### 5.2. Results of influencing factors

The results of the influencing factors are shown in Table 10. Nine influencing factors have a significant impact on the establishment of China's interprovincial low-carbon patent cooperation network. This indicates that the weighted degree distribution of China's interprovincial low-carbon patent cooperation network is affected by various factors, such as economic development, financial support, local scientific research level, and local awareness. The explanatory power of each influencing factor is between 40 and 70%. They can be divided into primary and secondary influencing factors according to the explanatory power. The primary influencing factors include the R&D expenditure of industrial enterprises above a designated size, the number of effective R&D invention patents of industrial enterprises above a designated size, and the low-carbon search index. The secondary influencing factors include per capita GDP, per capita disposable income of residents, value added of the secondary and tertiary industries as a percentage of GDP, local government fiscal expenditure as a percentage of GDP, science and technology expenditure as a percentage of local government fiscal expenditure, and the number of undergraduates and college students.

**Table 10:** Correlation coefficients between the weighted degree of the low-carbon patent cooperation network and influencing factors.

**Influencing factor**	**Detector**	**Correlation**	***P***
Quality of economic development	GDP per capita	0.5146	0.0733
	Per capita disposable income of residents	0.5463	0.0627
	Value added of the secondary and tertiary industries as a percentage of GDP	0.4657	0.0283
Financial support	Local government fiscal expenditure as a percentage of GDP	0.4333	0.0506
	Science and technology expenditure as a percentage of local government fiscal expenditure	0.5703	0.0570
Local scientific research level	R&D expenditure of industrial enterprises above the designated size	0.6022	0.0027
	Number of effective R&D invention patents of industrial enterprises above designated size	0.7155	0.0133
	Number of undergraduates and college students	0.4267	0.0404
Local awareness	Low-carbon search index	0.6801	0.0000

#### 5.2.1. Quality of economic development

The regression results showed a significant correlation with per capita GDP, per capita disposable income of residents, and value added of the secondary and tertiary industries as a percentage of GDP. This suggests that provinces with a reasonable provincial economic structure and high level of economic development can agglomerate to attract innovative elements and, thus, form a closer connection with other provinces for low-carbon patent innovation.

#### 5.2.2. Financial support

The regression results showed a significant correlation at the 10% level with local government fiscal expenditure as a percentage of GDP and science and technology expenditure as a percentage of local government fiscal expenditure. Moreover, a higher correlation was observed with science and technology expenditure as a percentage of local government fiscal expenditure, indicating that this factor plays the largest role in local financial support.

#### 5.2.3. Local scientific research level

The importance of science and technology is self-evident (42). Provinces with higher research capabilities have higher levels of low-carbon patent development. The R&D expenditures of industrial enterprises above a designated size and the number of effective R&D invention patents of industrial enterprises above a designated size ranked first and third, respectively, among all factors with significant correlations. This indicates that the local scientific research level has an important influence on the weighted degree of the low-carbon patent cooperation network.

#### 5.2.4. Local environmental awareness

The regression results showed a significant correlation at the 1% level with local low-carbon awareness. Moreover, it had a correlation coefficient of more than 0.6, ranking second among all influencing factors. This demonstrates that low-carbon awareness promotes local research and the application of low-carbon patents, thereby promoting low-carbon patent cooperation between localities.

## 6. Spatial evolution of the low-carbon patent cooperation networks of the five urban agglomerations along the eastern coast

As seen above, the eastern region contains the main provinces for low-carbon patent cooperation in China. Urban agglomeration is an important carrier for the connection and agglomeration of innovative elements. The five major urban agglomerations along the eastern coast are important growth poles for the high-quality economic development of China. Therefore, this study analyzes the network structure and influencing factors of low-carbon patent cooperation based on the five major urban agglomerations in eastern China.

### 6.1. Statistical characteristics

The basic statistical characteristics of the network were calculated in Gephi using the five urban agglomerations, i.e., BTH, YRD, PRD, SP, and WTS, as spatial units. As shown in Table 11, the network density of the five urban agglomerations increased significantly over time, with the patent cooperation between cities, especially within urban agglomerations, getting closer. In 2021, all cities in the urban agglomerations participated in the low-carbon patent cooperation network, except for the WTS. The low-carbon patent cooperation between BTH and the YRD was in the lead in terms of both network connectivity and connection strength.

**Table 11:** Statistical characteristics of the low-carbon patent cooperation networks of the five urban agglomerations along the eastern coast of China.

**Year**	**Urban agglomeration**	**Number of nodes**	**Number of edges**	**Network density**	**Average clustering coefficient**	**Average path length**	**Average degree**	**Average weighted degree**
2001	BTH	3	2	0.667	0.000	1.333	1.333	5.333
	PRD	2	1	1.000	0.000	1.000	1.000	1.000
	YRD	2	1	1.000	0.000	1.000	1.000	2.000
	SP	2	1	1.000	0.000	1.000	1.000	1.000
	WTS	2	1	1.000	0.000	1.000	1.000	1.000
2021	BTH	10	25	0.556	0.825	1.444	5.000	181.200
	PRD	9	21	0.583	0.826	1.417	4.667	122.444
	YRD	16	68	0.567	0.824	1.443	8.500	228.500
	SP	12	30	0.455	0.759	1.545	5.000	26.333
	WTS	8	10	0.357	0.761	1.375	2.500	24.000

### 6.2. Spatial pattern

As shown in Table 12, in 2001, no obvious core-periphery structure was observed in the urban agglomerations, except for BTH, where the low-carbon patent cooperation network showed a single-core structure with Beijing as the core. In 2021, the low-carbon patent cooperation networks in BTH, YRD, PRD, and WTS showed a single-core structure with Beijing, Shanghai, Guangzhou, and Xiamen as the core, respectively, and that of SP showed a dual-core structure with Jinan and Qingdao as the cores. Specifically, the low-carbon patent cooperation network within the PRD was based on the cooperation of Guangzhou and Shenzhen with Dongguan, Foshan, and Huizhou; cooperation within the YRD was based on the cooperation of Nanjing and Shanghai with Suzhou and Hangzhou; cooperation within BTH was based on the cooperation of Beijing with Shijiazhuang and Tianjin; that within the SP was based on the cooperation of Jinan and Qingdao with Yantai and Binzhou; and that within the WTS was based on the cooperation of Xiamen and Fuzhou with Zhangzhou and Quanzhou.

**Table 12:** Spatial pattern of interurban low-carbon patent cooperation networks in 2001, 2011, and 2021.

**Year**	**Urban agglomeration**	**Nodes (top 10 by weighted degree)**	**Edges (top 10 by weight)**
2001	BTH	Beijing (8), Tianjin (6), and Langfang (2)	Beijing-Langfang (2), and Beijing-Tianjin (6)
	PRD	Guangzhou (1) and Huizhou (1)	Guangzhou-Huizhou (1)
	YRD	Shanghai (2) and Nanjing (2)	Shanghai-Nanjing (2)
	SP	Qingdao (1) and Yantai (1)	Qingdao-Yantai (1)
	WTS	Xiamen (1) and Quanzhou (1)	Xiamen-Quanzhou (1)
2021	BTH	Beijing (823), Tianjin (370), Shijiazhuang (272), Baoding (137), Tangshan (53), Zhangjiakou (48), Chengde (35), Langfang (30), Cangzhou (28), and Qinhuangdao (16)	Beijing-Tianjin (325), Beijing-Shijiazhuang (224), Baoding-Beijing (108), Beijing-Tangshan (42), Beijing-Zhangjiakou (31), Beijing-Chengde (30), Beijing-Langfang (28), Beijing-Cangzhou (23), Baoding-Shijiazhuang (20), and Shijiazhuang-Tianjin (17)
	PRD	Guangzhou (396), Shenzhen (216), Dongguan (138), Foshan (108), Huizhou (82), Zhuhai (60), Zhaoqing (40), Jiangmen (38), and Zhongshan (24)	Dongguan-Guangzhou (91), Guangzhou-Shenzhen (89), Foshan-Guangzhou (58), Guangzhou-Zhuhai (57), Huizhou-Shenzhen (57), Dongguan-Shenzhen (34), Guangzhou-Jiangmen (31), Foshan-Shenzhen (28), Guangzhou-Zhaoqing (27), and Guangzhou-Huizhou (25)
	YRD	Shanghai (874), Nanjing (557), Hangzhou (520), Wuxi (506), Ningbo (415), Suzhou (286), Changzhou (100), Jiaxing (97), Nantong (67), and Zhenjiang (51)	Shanghai-Wuxi (408), Hangzhou-Ningbo (231), Shanghai-Suzhou (117), Nanjing-Shanghai (108), Nanjing-Suzhou (96), Hangzhou-Shanghai (90), Nanjing-Ningbo (90), Hangzhou-Nanjing (71), Ningbo- Shanghai (68), and Changzhou-Nanjing (56)
	SP	Jinan (81), Qingdao (81), Weifang (26), Weihai (24), Yantai (24), Dongying (22), Binzhou (18), Zibo (16), Dezhou (7), and Rizhao (7)	Jinan-Qingdao (29), Qingdao-Yantai (13), Binzhou-Jinan (12), Qingdao-Weifang (11), Qingdao-Weihai (10), Dongying-Qingdao (9), Dongying-Zibo (7), Jinan-Weihai (7), Jinan-Tai'an (6), and Dezhou-Jinan (5)
	WTS	Xiamen (66), Fuzhou (49), Zhangzhou (43), Quanzhou (17), Ningde (10), Putian (5), Shantou (1), and Chaozhou (1)	Xiamen-Zhangzhou (38), Fuzhou-Xiamen (21), Fuzhou-Quanzhou (10), Ningde-Fuzhou (9), Quanzhou-Xiamen (6), Zhangzhou-Fuzhou (5), Fuzhou-Putian (4), Quanzhou-Putian (1), Xiamen- Ningde (1), and Shantou-Chaozhou (1)

### 6.3. Betweenness centrality

The betweenness centralities of the five urban agglomerations are reported in Table 13. In 2021, Beijing, Guangzhou, Nanjing, Jinan and Fuzhou ranked first in terms of betweenness centrality, indicating their absolute leadership for the path of the low-carbon patent cooperation network and their core position in the network.

**Table 13:** Betweenness centrality of the nodes in the low-carbon patent cooperation network in 2021 (betweenness > 0).

**BTH**	**Betweenness**	**PRD**	**Betweenness**	**YRD**	**Betweenness**	**SP**	**Betweenness**	**WTS**	**Betweenness**
Beijing	9.83	Guangzhou	7.75	Nanjing	17.50	Jinan	20.08	Fuzhou	4.00
Tianjin	5.33	Shenzhen	3.75	Shanghai	11.00	Qingdao	8.92	Xiamen	1.50
Shijiazhuang	3.75	Foshan	2.25	Hangzhou	8.23	Weifang	3.33	Quanzhou	0.50
Baoding	0.83	Dongguan	1.25	Suzhou	5.40	Yantai	1.92		
Zhangjiakou	0.25			Ningbo	3.40	Zibo	0.67		
				Changzhou	2.77	Dongying	0.58		
				Wuxi	1.60	Weihai	0.25		
				Zhenjiang	1.60	Dezhou	0.25		
				Huzhou	0.33				
				Nantong	0.17				

### 6.4. Closeness centrality

In 2021, cities that ranked high in terms of closeness centrality in the low-carbon patent cooperation networks of the five urban agglomerations are Beijing, Tianjin, and Shijiazhuang in BTH; Guangzhou, Shenzhen, Foshan, and Dongguan in PRD; Nanjing, Shanghai, Hangzhou, and Suzhou in YRD; Jinan, Qingdao, and Weifang in SP; and Fuzhou, Shantou, and Chaozhou in WTS (Table 14). These cities occupied a prominent position in the network. This suggests that these nodes are closer to other innovative entities in the low-carbon patent cooperation networks of urban agglomerations in China. The shorter path length also enables these nodes to acquire network resources or search for partners in a shorter time and with greater efficiency than other nodes.

**Table 14:** Closeness centrality of the top 10 nodes in the low-carbon patent cooperation network in 2021.

**BTH**	**Closeness**	**PRD**	**Closeness**	**YRD**	**Closeness**	**SP**	**Closeness**	**WTS**	**Closeness**
Beijing	1.00	Guangzhou	1.00	Nanjing	1.00	Jinan	1.00	Fuzhou	1.00
Tianjin	0.90	Shenzhen	0.89	Shanghai	0.94	Qingdao	0.85	Shantou	1.00
Shijiazhuang	0.82	Foshan	0.80	Hangzhou	0.83	Weifang	0.73	Chaozhou	1.00
Baoding	0.75	Dongguan	0.80	Suzhou	0.83	Yantai	0.69	Xiamen	0.83
Zhangjiakou	0.69	Zhaoqing	0.67	Changzhou	0.79	Dongying	0.65	Quanzhou	0.71
Chengde	0.64	Jiangmen	0.67	Wuxi	0.75	Weihai	0.65	Zhangzhou	0.63
Qinhuangdao	0.64	Zhuhai	0.62	Zhenjiang	0.75	Zibo	0.61	Ningde	0.63
Tangshan	0.60	Huizhou	0.57	Ningbo	0.71	Binzhou	0.61	Putian	0.63
Cangzhou	0.56	Zhongshan	0.57	Huzhou	0.68	Dezhou	0.58		
Langfang	0.56			Nantong	0.65	Rizhao	0.55		

## 7. Influencing factors of the low-carbon patent cooperation network structure in urban agglomerations

### 7.1. Selection of influencing factors

The correlation between the weighted degree of the low-carbon patent cooperation networks of the urban agglomerations and the influencing factors was calculated with the weighted degree of the urban agglomeration networks in 2021 as the dependent variable and the number of patent applications, per capita GDP, low-carbon search index, the total volume of post and telecommunications business, and actually utilized value of foreign direct investment as independent variables. Since the 2021 Statistical Yearbook has not been published yet, data from 2020 were used for all independent variables, except for the low-carbon search index, for which data from 2021 were used.

### 7.2. Results of influencing factors

The gray relational analysis results in Table 15 show that the correlation and ranking of the influencing factors vary among urban agglomerations. The number of patent applications ranked second in terms of correlation in the YRD, SP, and BTH. This indicates that innovation capability has a strong correlation with the weighted degree of the interurban low-carbon patent cooperation networks in urban agglomerations. The total volume of post- and telecommunications business and per capita GDP generally ranked fourth or fifth, except that the former factor ranked first in the PRD and the latter ranked third in the WTS. The higher the levels of economic development and informatization, the greater the possibility of low-carbon patent cooperation. The low-carbon search index represents local awareness of low-carbon development. This influencing factor ranked highest in terms of correlation in the YRD, SP, and BTH and ranked second in the PRD and WTS. This demonstrates the importance of low-carbon development awareness to low-carbon patent cooperation in urban agglomerations. The utilized value of foreign direct investment represents a city's level of technology imported from overseas through foreign direct investment. Foreign investment can quickly drive local industrial development and promote urban industrial transformation and development, thereby promoting local low-carbon patent cooperation.

**Table 15:** Correlation between the weighted degree of nodes in the low-carbon patent cooperation networks of the five urban agglomerations along the eastern coast of China and the influencing factors.

**Influencing factor**	**PRD**		**YRD**		**SP**		**BTH**		**WTS**	
	**Correlation**	**Rank**	**Correlation**	**Rank**	**Correlation**	**Rank**	**Correlation**	**Rank**	**Correlation**	**Rank**
Number of patent applications	0.736	3	0.787	2	0.808	2	0.865	2	0.858	4
GDP per capita	0.729	4	0.640	5	0.654	5	0.731	4	0.869	3
Low-carbon search index	0.782	2	0.800	1	0.834	1	0.899	1	0.888	2
Total volume of post and telecommunications business	0.785	1	0.769	4	0.698	4	0.682	5	0.815	5
Actually utilized value of foreign direct investment	0.719	5	0.784	3	0.718	3	0.855	3	0.896	1

## 8. Conclusions and implications

### 8.1. Conclusions

The present study analyzes the number of low-carbon patent applications and the structural characteristics of the cooperation networks in China at the provincial and urban agglomeration levels using social network analysis based on data from the Incopat global patent database since 2001; it reveals the climate governance pattern established in China to achieve the goals of peak carbon emissions and carbon neutrality. The following conclusions were reached.

The total number of low-carbon patent applications in China increased annually. Specifically, two characteristics are present in the number of low-carbon patent applications in China due to local economic development, scientific research, and policies. First, there were great changes in the number of low-carbon patent applications. Second, the number of low-carbon patent applications varied significantly between provinces, but such regional differences have been decreasing.

In terms of provincial cooperation, China has formed an effective low-carbon cooperation network as a whole, showing obvious spatial correlation and spillover effects and increasing clustering. In terms of individual network characteristics, Beijing, Jiangsu, Guangdong, Shanghai and other provinces along the eastern coast occupy a core position in the network and play an important role in utilizing structural holes and bridging. In terms of segmentation, three types of blocks have been formed: bidirectional spillover, main benefit, and broker blocks. There is a clubbing effect between the blocks. In terms of regional connections, the interregional low-carbon patent cooperation network is not only more closely connected but also has stronger connection strength than the intraregional interprovincial cooperation network, except for east-east cooperation.

In terms of urban agglomeration, the size of the low-carbon patent cooperation networks of all five urban agglomerations increased significantly over time, with the interurban patent cooperation getting closer. The cooperation within the urban agglomerations showed an integrated radial pattern centered on the central city of the urban agglomeration. GRA reveals that urban innovation capability, economic development, low-carbon development awareness, level of technology import from overseas, and informatization level are highly correlated with the weighted degree of the low-carbon cooperation network.

### 8.2. Implications for public health

At present, China is in a critical period of low-carbon transformation. In addition, it may be difficult to control the global impact of the COVID-19 pandemic in a short period of time. Therefore, local governments need to combine local economic development with low-carbon development and integrate low-carbon development into all aspects of local development (43). Accordingly, the following suggestions are proposed. First, the COVID-19 pandemic has restricted and affected exchanges and cooperation between regions to varying degrees. Therefore, efforts should be made to explore the potential for cooperation between patent applicants in the low-carbon field. By developing technical information-sharing platforms, promoting urban digitalization, and establishing incentive mechanisms, the low information transmission efficiency caused by geographic and technological distance can be overcome, and new cooperation can be established between patent applicants on an ongoing basis, thereby expanding the breadth of cooperation and exploring the potential for cooperation within the low-carbon patent cooperation network.

Second, important provinces or cities in the low-carbon patent cooperation network should be encouraged to play a leading role. While making every effort to prevent COVID-19, core provinces or cities should be encouraged to carry out further cooperation (44). Moreover, efforts should be made to facilitate low-carbon patent cooperation between the core and peripheral provinces or cities under the leadership of the core provinces or cities. In this way, the internal resource allocation of the network can be optimized, the sustainable development of the low-carbon industry promoted, the bridging role of universities and research institutes further enhanced, and the resources of enterprises fully utilized.

Third, actions should be taken to establish cross-regional cooperative groups for low-carbon patent development. The burden of the COVID-19 pandemic on the economically underdeveloped central and western regions is self-evident. Therefore, efforts should be made to fully exploit the technical advantages and economic assistance of industry-university-research organizations in the eastern provinces, cities, and urban agglomerations, promote the interconnections and interactions between the eastern and central regions, and strengthen the leading role of the east for the west. This will contribute to achieving cross-regional low-carbon technology cooperation and establishing a reasonable spatial pattern for promoting the development of low-carbon technologies for energy conservation and environmental protection in China.

Finally, more financial support should be provided to increase R&D investment and efforts, especially for the R&D of low-carbon technologies. Local governments should seize the development opportunities brought by the COVID-19 pandemic and devote great efforts to developing low-carbon technologies to achieve corner overtaking in regional development.

### 8.3. Theoretical contributions

First, this study attempts to investigate China's low-carbon patent cooperation network at the national, urban agglomeration, and regional levels, employs the existing network analysis method in a vertical three-dimensional manner, and reveals the pattern characteristics of the low-carbon patent cooperation network in different dimensions. It breaks the limitation of the single-dimensional perspective of traditional network research and expands the theoretical system of the geography of innovation.

Second, this study analyzes the position and role of each node city in the innovation networks at the urban agglomeration, regional, and provincial levels, which not only helps identify the innovation functions and roles of cities but also facilitates the deepening of the theoretical research of innovative cooperation between cities.

### 8.4. Limitations and directions for further research

Despite its contribution, this study has some limitations. First, this study is limited to China and did not investigate the low-carbon patent cooperation between countries in the context of globalization. Second, the influencing factors selected in the study are not comprehensive enough due to limited data availability. In future research, the mechanisms that affect the low-carbon patent cooperation network can be further explored through other methods, such as field surveys, and further empirical studies of the influencing factors using econometric methods. Third, due to space limitations, the spatial pattern and influencing factors of the national low-carbon patent cooperation network have not been analyzed at the level of prefecture-level cities. Further research could also be conducted on these aspects.

## Data availability statement

The original contributions presented in the study are included in the article/supplementary material, further inquiries can be directed to the corresponding author.

## Author contributions

All authors listed have made a substantial, direct, and intellectual contribution to the work and approved it for publication.
